# Engineered cells that sense and respond to interleukin-23 with neutralizing therapy for autoimmunity

**DOI:** 10.64898/2026.10.12.758578

**Published:** 2026-10-14

**Authors:** Gabriela L. Garza, Sonja N. L. Danon, Jonathan Wuchter, Aaron H. Morris

## Abstract

Engineered cell sensors are powerful tools for monitoring biomarkers and delivering timely therapy. Here, we create an engineered cell sensor for interleukin-23 (IL-23), a critical biomarker in many autoimmune diseases, and a therapeutic delivery platform for autoimmune disease management. We utilized Modular Extracellular Sensor Architecture (MESA), a promising platform given its modularity and previous use to developed cell sensors for immune targets such as IL-10. First, we developed a library of IL-23 MESA receptors by incorporating IL-23-specific nanobody binders and performed high-throughput functional screening. We achieved further receptor optimization by modifying the extracellular linker length and the ratio of receptor chain expression. Receptor performance was highly correlated with predicted geometric features such as binder pair distance and degree of transmembrane domain dimerization. The receptors were limited by poor surface expression and functioned most effectively as sensors for intracellular IL-23. Finally, we modified the IL-23 sensing circuit to output an IL-23 neutralizing nanobody with the potential to reduce inflammation triggered by IL-23 secretion. This sense-and-respond architecture can be utilized for dynamic therapeutic treatment to meet the dynamic relapse-remitting pattern of many autoimmune diseases.

**Visal Abstract:**

